# Towards optimising experimental quantification of persistent pain in Parkinson’s disease using psychophysical testing

**DOI:** 10.1038/s41531-021-00173-y

**Published:** 2021-03-17

**Authors:** Kirsty Bannister, Rory V. Smith, Patrick Wilkins, Tatum M. Cummins

**Affiliations:** 1 Central Modulation of Pain, Institute of Psychiatry, Psychology and Neuroscience, King’s College London, London, UK; 2 Department of Population Health Sciences, School of Population Health & Environmental Sciences, King’s College London, London, UK

**Keywords:** Parkinson's disease, Predictive markers

## Abstract

People with Parkinson’s disease (PD) may live for multiple decades after diagnosis. Ensuring that effective healthcare provision is received across the range of symptoms experienced is vital to the individual’s wellbeing and quality of life. As well as the hallmark motor symptoms, PD patients may also suffer from non-motor symptoms including persistent pain. This type of pain (lasting more than 3 months) is inconsistently described and poorly understood, resulting in limited treatment options. Evidence-based pain remedies are coming to the fore but therapeutic strategies that offer an improved analgesic profile remain an unmet clinical need. Since the ability to establish a link between the neurodegenerative changes that underlie PD and those that underlie maladaptive pain processing leading to persistent pain could illuminate mechanisms or risk factors of disease initiation, progression and maintenance, we evaluated the latest research literature seeking to identify causal factors underlying persistent pain in PD through experimental quantification. The majority of previous studies aimed to identify neurobiological alterations that could provide a biomarker for pain/pain phenotype, in PD cohorts. However heterogeneity of patient cohorts, result outcomes and methodology between human psychophysics studies overwhelmingly leads to inconclusive and equivocal evidence. Here we discuss refinement of pain-PD paradigms in order that future studies may enhance confidence in the validity of observed effect sizes while also aiding comparability through standardisation. Encouragingly, as the field moves towards cross-study comparison of data in order to more reliably reveal mechanisms underlying dysfunctional pain processing, the potential for better-targeted treatment and management is high.

## Introduction

Parkinson’s disease (PD) is a progressive, chronic and complex neurodegenerative disease affecting 6.1 million people worldwide^1^. While the aetiology of PD is not well understood a number of key studies have contributed to our understanding of the development of sporadic as well as familial PD^2–7^. The pathogenesis of PD involves the degeneration of dopaminergic neurons in the substantia nigra pars compacta, causing progressively diminished dopamine synthesis in the striatum. Diagnosis of PD follows physical examination of the motor symptoms of the disease, including tremor and postural instability, which appear once 50–70% of nigrostriatal neurons are depleted^8,9^. However, there is a significant loss of dopaminergic neurons during the prodromal phase, which is reported to last several years before motor symptoms manifest^10^. Although equally bothersome, the non-motor symptoms of PD are poorly recognised and underreported^11,12^ placing a significant burden on affected individuals’ quality of life^13–15^. Persistent pain is a particularly problematic non-motor symptom affecting up to 85% of individuals with PD^16,17^ and epidemiological studies highlight the need to systematically investigate pathophysiology based treatment strategies^18,19^.

### Pain in PD: Current treatment

Pain is a multi-dimensional experience involving sensory discriminative and affective motivational descriptive axes. As such, pain perception is inherently subjective and influenced by multiple factors. While acute pain reflects an adaptive survival mechanism, persistent pain negatively impacts the quality of life of the affected individual and serves limited evolutionarily advantage. Unfortunately a large proportion of people with PD experience persistent pain and 50% of those individual’s receive no or inadequate treatment^20,21^. Therapeutic strategies that offer an improved analgesic profile remain an unmet clinical need. Clearly multi-disciplinary approaches for pain management that encompass new concepts in pathogenesis and treatment are required^22,23^.

The optimisation of dopaminergic therapies is generally accepted as a first step in the current clinical management of persistent pain in PD^24,25^. PD patients report more pain when ‘off’ Levodopa^26,27^ and painful sensations are ameliorated (though not eliminated) ‘on’ Levodopa^28^. Dopamine receptor agonism has shown promise. The RECOVER and DELORES studies, a pair of double blind, placebo-controlled trials, support the analgesic role of a rotigotine transdermal patch in PD^29,30^ while the multi-center, observational, open-label EUROINF study advocated the use of the apomorphine in improving the non-motor symptoms (NMS) scale, which includes a measure of pain^25,31^. Other therapies that have been explored include oxycodone-naloxone and duloxetine^32,33^. However despite progress in the field a recent Movement Disorders Society Task Force cited only two evidence-based pain treatment options^34^. Increasingly it is recognised that pathophysiologies may represent non-dopaminergic mediated effects^35^. Understanding why current treatment options are limited (and why those available are largely ineffective) is straightforward when considering that not only is pain multifactorial in origin, but also that the precise underlying mechanisms responsible for the initiation and maintenance of persistent pain in PD are not fully understood. Put broadly, the experience of pain is unique according to the individual and their pain type.

### Pain in PD: Assessment and classification

It is vital that the pain type experienced by the affected person with PD is explicitly and correctly assessed and classified in order that targeted therapies can be offered. Coupled with an elusive driving force (mechanistically speaking), a lack of consensus regarding the appropriate assessment and classification of pain in PD patients has thwarted analgesic success. In pain assessment terms, the ‘King’s Parkinson’s Disease Pain Scale’ (KPPS)^36^ is advocated by the ‘International Parkinson’s and Movement Disorder Society Non-Motor PD Study Group’ for the evaluation of pain in PD. Encouragingly, an international multi-center validation study reported a strong correlation between the KPPS and quality of life scores, as well as excellent inter-rater and test-retest reliability^36^. Heralded as a novel approach for the assessment of pain in PD, the KPPS, while lengthy for routine clinical care, allows in-depth profiling in clinical trials based on the subjective reporting of an individual’s pain experience. Regarding pain classification, the Ford criteria^26^ classified PD-related pain into five groups: musculoskeletal, neuropathic/radicular, central/primary, dystonic and akathisia. The underlying mechanisms causing, for example, musculoskeletal versus central pain are very different in sensory terms and thus the analgesic regimen should be also. Understanding the complexities of pain processing pathways allows one to highlight and explain the heterogeneity of analgesic success with agents including Levodopa and dopamine receptor agonists.

In order that novel and optimal pharmacotherapeutic targets may be identified, a deeper understanding of the pain circuitry in PD patients who experience persistent pain is required. Since we know that unique maladaptive changes occur in central modulatory pathways that govern pain and neurodegeneration, identifying pathophysiological hallmarks for persistent pain and Parkinson’s disease states, likely highly plastic and stage specific, is crucial.

## Experimental assessment of pain

Psychophysical assessment is a clinical research technique that can improve our understanding of the mechanisms underlying the development of persistent pain. The ultimate goal of ‘personalised pain management’ requires a thorough understanding of the neuronal mechanisms that contribute to persistent pain in a given patient^37,38^. Temporal summation (TS), quantitative sensory testing (QST) and conditioned pain modulation (CPM) paradigms are three examples of tests that are psychophysical in nature as they assess the perception of pain via salience circuitry^39^, as opposed to the subconscious processing of stimuli, which may or may not reach conscious perception^40^. As such, psychological factors such as anxiety^41^, depression^42^ and cognitive factors including attention and anticipation^43^ can influence pain perception during psychophysical testing. A recent review beautifully summarises the peripheral and central changes induced in chronicity as well providing a coherent schematic representation of psychophysical paradigms used in experimental pain research settings^44^. Human psychophysics paradigms may control for the influence of these confounders by demanding test-retest paradigms in order that it be possible to unpick the fluctuating nature of each individual’s sensory profile when analysing data from multiple sessions. It is vital to acknowledge that, even when using human psychophysics testing, it is not possible to pinpoint where precisely in the pain neuraxis a dysfunction is (the pain percept being an entirely central construct). A focal measure of, for example, peripheral versus spinal malfunction, cannot be made.

### Static and dynamic psychophysical paradigms

So-called static psychosocial paradigms refer to a range of quantitative sensory testing (QST) protocols, which were recently standardised and defined by the German research network on neuropathic pain (DFNS)^45^. In addition to sensory detection thresholds the DFNS protocol involves pain thresholds to thermal (cold and heat) and mechanical (pressure and pinprick) stimuli. If QST responses are incongruous to normative reference values (i.e. refs. ^45,46^.) the dysfunction may be located anywhere along the neural axis, from peripheral nerve fibres^47,48^, to the spinal cord^49^ and cortical areas^50^. However the value of QST to distinguish central and peripheral mechanisms is limited. Nociceptive withdrawal reflex (NWR) thresholds offer a measure of central pain processing, specifically spinal nociceptive facilitation^51^.

Dynamic psychophysical paradigms are believed to better define central mechanisms of pain processing compared to static paradigms^52^. For example CPM paradigms measure the functionality of the descending pain-inhibitory system. Interestingly, CPM is the human correlate of the diffuse noxious inhibitory control effect observed in animals where the ‘pain inhibits pain’ phenomenon is activated upon application of a testing stimulus concurrent to a conditioning stimulus^53,54^. Evidence from translational studies suggests that CPM reflects the functionality of pain-modulating brainstem regions in inhibiting the activity of spinal neurons^55,56^. In contrast, pain facilitation mechanisms can be assessed through TS paradigms where pain responses to single noxious stimuli are compared with frequency-dependent responses to serially presented (identical) noxious stimuli^57^.

### Sensory profiling and the potential for mechanism-based treatment

Since psychophysical testing offers the opportunity to explore the functionality of an individual’s pain system under controlled settings, a comprehensive assessment of various pain processing and modulatory pathways for use as a surrogate measure of the mechanisms driving the development of persistent pain in a given population/patient cohort is possible^38^. For example CPM deficiencies in patients with neuropathic pain can be targeted by manipulation of central noradrenergic and serotonergic transmission, where the pain-inhibiting impact of Tapentadol (noradrenaline reuptake inhibitor and μ-opioid receptor agonist) potentiates impaired CPM in persistent pain patients in a manner that back translates to animal studies^53,58,59^. Psychophysical testing can also be used to predict analgesic treatment efficacy^60^.

In people with PD, sensory profiling through psychophysical testing has been applied in order to provide insight of the underlying mechanisms of persistent pain. Thereafter, guidance for personalised pain medicine through mechanism-based treatments is a key goal for many chronic pain types^61–64^. However, a frustratingly disparate range of psychophysical trials exists in the literature for the PD patient cohort, where significant differences in the type of pain considered and methodologies employed leads to incomplete conclusions, as discussed below.

## Psychophysical testing in people with PD

It is documented that PD patients have hyperalgesic responses upon psychophysical testing compared to healthy controls^65–73^. Two recently published systematic reviews found the differences in PD patients’ sensitivity to pain to be significantly different from non-PD populations^74,75^. However, the data are inconsistent^43,76–79^ and considerable clinical heterogeneity and methodological inconsistencies throughout the literature limits comparison between studies and thus the clinical applicability of the findings. In total (1) failure to control for the clinical heterogeneity of people with PD including the correct characterisation of the pain type being assessed, and (2) methodological inconsistencies when performing the experimental quantification of pain impairs reliability between studies and contributes to the contradictory findings in the PD pain psychophysics literature. A lack of standardised pain definitions has led not only to result-impacting differences in inclusion and exclusion criteria, but also to result-impacting differences in the pain outcomes assessed. Additionally, on the whole, previous studies appear often underpowered, open label and missing relevant comparator groups. Future studies should include larger sample sizes and standardised pain classification (for example the KPPS) and methodological approaches in order to investigate the aetiology underlying different types of pain in PD, as each have unique mechanisms that need to be thoroughly understood in order to establish individualised therapeutic intervention.

### Clinical heterogeneity

One of the major issues with psychophysical testing in terms of pain studies in PD patient cohorts is that, despite a high prevalence of persistent pain, many participants tested do not actually suffer from persistent pain^77,80–82^. Worse still, patients with and without persistent pain may be grouped into one PD patient group^80,81^. In studies where people with PD with and without persistent pain are segregated, a lack of appropriate pain classification means that identification of whether pain status is the driver of potential altered psychophysical responses in comparison to healthy volunteers is complicated. As such, the substantial clinical heterogeneity within cohorts of PD patients influences psychophysical responses differentially leading to equivocal conclusions.

The presence and categorisation of clinical pain, prescription of levodopa, disease severity, age, PD sub-types and symptomatic laterality (i.e. unilateral or bilateral), differed among the trials that were researched during our literature search. Regarding persistent pain characterisation, undefined clinical pain was often a criteria for exclusion^83–85^, or not reported^79,86^. This is an issue when considering that it is well established that the presence of clinical pain, both persistent and acute, influences the way in which an individual reports their pain perception^87,88^. It is also a problem when considering that the pain type experienced by people with PD may influence the effect of dopaminergic medication, evidenced by Levodopa worsening dystonic pain^89^ but improving musculoskeletal pain^90^. The influence of Levodopa on psychophysical responses has been investigated in PD populations, but with conflicting results^49,72,91^. A meta-analysis by Thompson et al.^75^, revealed that pain threshold values in PD patients were significantly attenuated following Levodopa administration, suggesting that dopamine deficient states may contribute to hyperalgesia. However, interpretation of the literature is limited due to the variable nature of the methodologies utilised (see section below) and results reported, where the paramount concern regarding consistent pain assessment and classification was not considered between studies. The inconsistent reporting of pain type or even presence of persistent pain contributes to inconsistencies within the evidence, and standardised reporting and classification of pain is imperative if valuable conclusions are to be drawn. Several studies directly investigated the influence of clinical pain on psychophysical responses by comparing PD patients with pain against those without. Most reported that the presence of pain increased pain sensitivity^33,92–94^. However, several dispute these findings, reporting the presence of pain had no such influence^72,73,76,95,96^. These inconsistencies may be in part explained by the low statistical power of the trials as, of the studies that report clinical pain had no influence on psychophysical responses, only one included a cohort of more than 15 PD patients with pain^74^. Small sample sizes are confounded by considerable variability of pain characteristics across those studies cited, including primary central pain, musculoskeletal pain with dystonia, mixed non-dystonic, or undescribed pain. The inconsistent classification of pain is likely due to a previous lack of consensus regarding the appropriate assessment and classification of pain in PD^97^.

### Methodological inconsistencies

Identifying whether or not there is a difference in QST, TS and/or CPM responses in people with PD with persistent pain is hampered by the fact that, for many of the human psychophysical paradigms used, there is no consensus on the gold standard methodological approach. This is especially an issue when considering CPM paradigms where the variability between testing and conditioning stimuli is high. Previous evidence of a negative correlation between PD severity and CPM responses^77^, and a high prevalence of persistent pain in late-stage PD^98^, suggests that, as with other chronicities^58,99,100^, impaired descending pain modulation may develop early in PD, and worsen with disease progression. However few clinical trials have investigated CPM functionality in PD patients and no significant difference in CPM response between PD patients and healthy controls has been reported in four studies^69,77,80,96^. However, the testing and conditioning stimuli varied between the studies and only one study used an individually calibrated conditioning stimulus^69^. This latter point is a vital consideration if only acknowledging the fact that pain perception is inherently subjective, so what is threshold noxious to one individual may be intolerable to another. A considerable methodological limitation, evidenced principally by the failure in all but one study^61^ in successfully eliciting a CPM response in healthy control subjects, means that no conclusions regarding the functionality of the endogenous descending pain-inhibitory pathways in people with PD can even be drawn; it should not be possible to state that CPM functionality is maladaptive in PD patients if functionality cannot be demonstrated in the healthy population with the paradigm applied.

The major variable factor with QST studies is the method by which thresholds are determined, and therefore some are vulnerable to overestimation of pain thresholds^101^. When reviewing case controlled QST studies to investigate pain in a PD population, only one used the standardised DFNS protocol^76^. Additionally, control for dopaminergic medication was inconsistent with one assessing QST in drug naïve patients^76^. Exclusion criterias were generally consistent throughout the literature with some notable variation. Depression was screened for a handful of studies with some using tools that have been previously validated for screening in a PD population^102^ while two studies did not exclude patients who suffered from chronic pain unrelated to PD^68,72^. While assessment of PD pain was completed with the Ford classification in six studies, an informal pain assessment was completed in two others^72,103^ and pain was not classified at all in one^81^. Pain status was used to compare outcomes in several studies^68,69,72,76,92^. No effect of pain status was found on pain thresholds, except for one electrical pain threshold outcome^92^.

## Mechanisms that contribute to persistent pain in PD

As the field progresses psychophysical testing has the potential to advance our understanding of persistent pain in PD by elucidating the mechanisms which underlie pain in PD, and in doing so, identifying subgroups of patients with susceptibility to developing persistent pain while assisting in the development and monitoring of personalised pain management strategies for these patients.

### Initiation, propagation and maintenance of the pain state

While we do not know the underlying mechanisms that drive PD singular, persistent pain singular, nor persistent pain in PD, bench and bedside research investigative efforts have partially defined some of the factors important in the initiation, propagation and maintenance of each. Continued forward and back translational preclinical and clinical research will provide comprehensive disease pathology insight and guide towards a mechanism based (as opposed to a disease based) therapeutic approach to facilitate analgesic target identification. Psychophysical testing in humans, with its promise to link animal and clinical pain studies, is essential to fully understand the mechanisms that contribute to the development of persistent pain.

Hyperalgesic responses in PD may be attributed to excitability in dorsal horn neurons, evidenced by enhanced facilitatory responses to noxious stimuli in TS and nociceptive withdrawal (NWR) paradigms. Enhanced spinal nociception with reduced NWR thresholds to electrical stimuli was found in PD patients with pain^69^ and without pain^49^ indicating that the central sensitisation and facilitation of nociceptive inputs may contribute to hyperalgesic psychophysical responses in PD^71,75^. In addition, several studies report significantly increased sensitivity to the TS of pain in PD patients compared with controls^65,66,70,71^. Cumulatively these data suggest that a functional enhancement of nociceptive transmission could mediate persistent pain in PD. Enhanced pain responses may be consequential to impairments within supraspinal pain-modulating pathways as dysfunction in striatal adreno-dopaminergic inhibitory projections to the dorsal horn have been shown to result in inefficient attenuation of neuronal responses^104^ and are impaired in several persistent pain conditions including PD^105–107^. Functional magnetic resonance imaging (fMRI) techniques also reveal higher activation of somatosensory brain regions in PD patients compared with healthy controls^43,65,83^. While discussion of fMRI studies in PD is beyond the scope of this review, cumulatively the data indicate abnormal central nociceptive processing and central sensitisation, which may contribute to hyperalgesic psychophysical responses and the development of persistent pain in PD.

### Pathological links between PD and persistent pain

Do central processing abnormalities act as a catalyst for developing persistent pain in PD? And are they linked to those acting as a catalyst for the development of PD itself? We know that maladaptive central nervous system plasticity underlies the aetiology of PD, while multiple lines of evidence demonstrate that one important mechanism underpinning varied persistent pain states is maladaptive plasticity in central descending inhibitory pathways. Unique descending inhibitory pathways, including diffuse noxious inhibitory controls (DNIC), are sub-served by monoaminergic neurotransmission^53,108^, and monoaminergic neurotransmission is affected by PD-specific neurodegenerative changes already at the prodromal stage of the disease^109^. It is possible that there is a link between an underlying mechanism of PD and the development of persistent pain, where an established link could be therapeutically targeted thus improving not only the level of pain experienced by the affected individual, but also PD progression. Performing the appropriately powered human psychophysics pain experimental quantification studies would have the potential to contribute to our understanding of how the nervous system acts endogenously to modulate pain perception in PD, reveal whether this is linked to the aetiology of PD, and therefore unveil targets for intervention in the management of chronic pain in a personalised manner.

## Recommendations for future research

### Standardised testing and powered cohorts

Future research should control for confounding factors by standardising variables across laboratories. For example, the presence of pain should be classified according to an internationally validated scale, e.g. the KPPS. PD sub-types should be standardised according to an internationally verified method. Although there is no gold standard for sub-type classification, distinctions have been made between tremor-dominant and non-tremor-dominant sub-types^110^, and by using UPDRS-III sub-type-based classifications^111^. It is recommended that future studies should classify sub-types according to the German AWMF guidelines (i.e. tremor-dominant, akinetic-rigid and mixed-type sub-types, as this is the most frequently reported categorisation in the PD literature^76,80,96^. In addition, poor blinding of assessors was prevalent throughout the literature, an issue not limited to PD studies but apparent throughout the pain psychophysics literature. It is recommended psychophysical studies utilise the QAREL checklist^112^ to ensure methodological quality and diagnostic reliability. And finally, the majority of studies were underpowered to assess pain due to inadequate sample sizes, meaning drawing statistically reliable conclusions was not possible. Future studies should include large sample sizes and be conducted across multiple centres. A Bayesian statistical approach would represent an appropriate way to provide deeper insights into potential group differences (i.e. between pain types, among sub-types, etc.).

## Conclusion

Nigrostriatal degeneration and Lewy body pathology in key structures involved with pain perception and modulation may predispose individuals with PD to the development of persistent pain early in the prodromal phase. This is evident clinically as persistent pain often precedes the cardinal motor signs of the disease. Pain sensitivity scales with PD severity with psychophysical pain thresholds and CPM responses negatively correlated with disease progression, likely contributing to the observed high prevalence of pain in late-stage PD. Overlapping pathophysiological mechanisms are common to the development of the two disease states (persistent pain and Parkinson’s) and this becomes more clinically and behaviourally relevant as the disease progresses. Psychophysical testing is a crucial clinical investigative technique that has the potential to advance our understanding of pain in PD and reach the goal of personalised pain management by (1) elucidating the mechanisms that underlie pain in PD, (2) identifying subgroups of patients susceptible to developing persistent pain and (3) assisting in the prescription and monitoring of mechanism-based neurotherapeutic treatment in these patients. A lack of standardisation amongst laboratories limits the comparability of studies and is a major drawback for understanding the relevance of these paradigms in relation to dysfunctional mechanisms that contribute to pain in PD. There is an urgent need for an internationally agreed definition of pain in PD and a universally agreed consensus on protocols to perform dynamic psychophysical testing. Clarity in this regard will expedite the process of improved analgesic outcomes for those affected individuals.
